# The emotional intelligence of pediatric residents – a descriptive cross-sectional study

**Published:** 2017-02-24

**Authors:** Scott A. McLeod, Lyn K. Sonnenberg

**Affiliations:** 1 Department of Pediatrics, Faculty of Medicine and Dentistry, University of Alberta, Alberta, Canada; 2 Section of Developmental Pediatrics, Alberta Children’s Hospital, Alberta, Canada

## Abstract

**Background:**

Emotional Intelligence (EI) is a type of social intelligence. Excellent scores are achieved by displaying high levels of empathy in interpersonal relationships, strong skills in managing stressful situations as well as other personal competencies. Many of the social competencies that EI describes may have a direct impact on patient care. The objective of this study was to describe EI of pediatric residents and to identify if there are EI skills that should be selected for targeted intervention.

**Methods:**

This was a cross-sectional study administering the EQ-i 2.0© psychometric instrument to pediatric residents at the University of Alberta.

**Results:**

Thirty-five residents completed the EQ-i 2.0© (100% response rate). Their overall EI score was not significantly different than a normative group of college-educated professionals. Residents had relative strengths in the subcategories of *Emotional expression, Interpersonal Relationships, Empathy*, and *Impulse Control* (all p<0.05). Areas of relative weakness were in the subcategories of *Stress Tolerance, Assertiveness, Independence,* and *Problem Solving* (all p<0.05)*.*

**Conclusion:**

The EI of pediatric residents is consistent with that of other professionals. Educational interventions may be useful in the areas of weakness to enhance the physician-patient relationship.

## Introduction

Emotional intelligence (EI) is a type of social intelligence that is based on a number of facets including the ability to monitor and adapt to one’s own emotions, the degree of empathy that one displays in interpersonal relationships, the flexibility to adapt to change that one exhibits, and one’s ability to manage stressful situations.^1^,^2^

The first model of EI was proposed by Mayer and Salovey in 1990.^3^ They had initially conceptualized EI to contain three major components: 1) the ability to appraise the emotions of oneself and others; 2) the ability to regulate emotion; and 3) the ability to utilize emotion to facilitate various activities.^1^ Subsequently, other models of EI were proposed by Bar-On (1997)^4^, Goleman (1995)^5^, and most recently Petrides (2007)^6^. There are many common components to the models with recurring themes including awareness of one’s own emotions, empathy toward others, and adaptability.^7^

Within medicine, there has been an explosion of research into EI since many of the personal, emotional, and social competencies described within each of the models may have a direct impact on patient care and practice.^8^–^10^ EI-related abilities or personality traits are considered to be central tenets of the physician-patient relationship.^11^ Strong capabilities in the recognition and appraisal of patient emotions, and the harnessing of one’s empathy to tailor recommendations for individual patients, is patient-centered and may also lead to a better therapeutic alliance and decreased medical costs in our already over-burdened health system.^7^,^11^–^13^

Within our study, we utilized the EQ-i 2.0© model, which is based on the original Bar-On (1997)^4^ model. Within this model, there are composite categories (self-perception, self-expression, interpersonal, decision making, and stress management) that contain within them subcategories that in turn encompass some competencies commonly referred to as useful in the practice of medicine (see Table 2 for list of composite and subcategories). For example, high scores for *Emotional Expression* may imply that residents are comfortable in speaking about how they feel and are not afraid to make gestures of emotion, such as sharing happiness or sadness with patients and their family members. Successful relationships with patients and their families can grow from these exchanges of emotion and also can be useful in resolving conflict in the workplace. High scores in *Empathy* may mean a high degree of sensitivity and respect which would be beneficial in working with ill children that have complex needs. Strength in the *Interpersonal Relationships* category should facilitate open and trustworthy relationships between team members and patients alike. High scores in the category *Impulse Control* may indicate deliberation regarding a situation before acting and consideration of the viewpoints of others.

Enthusiasm for the study of EI has led to many different studies that have included residents: General Surgery, Orthopedics, Internal Medicine, Pathology, and Anesthesia.^14^–^17^ Studies of pediatric residents are limited to small samples in two recent studies, which also combined medicine and pediatric trainees.^16^

The purpose of this study was to describe the EI of general pediatric residents and to identify any EI skills that should be selected for targeted intervention in this population. Due to the nature of the pediatric practice relying on patient and family-centeredness as well as the sensitive nature of discussing the treatment of children with parents, we hypothesized that the overall EI of pediatric residents would be above the population average and above the EI previously reported for surgical residents. We did not expect to find a significant difference in EI related to the number of years of residency training since a previous study found no such difference.^15^ In terms of gender differences, we expected that women would outperform men in some subcategories such as *Empathy* and *Interpersonal Relationships* with men scoring higher in *Decision Making* and *Stress Tolerance,* similar to trends present in the general population.^15^ We also expected that EI would increase with age as has previously been reported.^16^,^18^

## Methods

All residents in the University of Alberta general pediatric residency program were invited to complete the EQ-i 2.0©, a well validated EI-measurement instrument, in March 2015. The EQ-i 2.0© is an online psychometric instrument which can be completed in 20–30 minutes.^18^ It was chosen over the other available EI measurement tools since its previous version had been used in similar studies with other resident groups.^15^,^19^,^20^ There are 133 items on this self-assessment and the questions employ a 5-point Likert scale. The test reports an overall EI score, which is also broken down into five composite scores and 15 subcategory scores. All of the scores are normalized to a mean of 100. The test was validated with normative groups including members of the general population and a sample of professionals (individuals with, at minimum, a college education who were employed or self-employed).^18^ A standard sample of professionals (n=1400) scored 8.6 points higher on the EQ-i 2.0© when compared to a general population normative group of 4000 individuals (p>0.0001).^18^

This study was approved by the Health Research Ethics Board at the University of Alberta. Consent was implied by submission of the EQ-i 2.0©. Participants received individual feedback worksheets with their EI results as incentive for participating. No other incentive was provided. Participation was voluntary and completing the survey was not part of the post graduate training curriculum.

The data were analyzed by year of training (Post Graduate Year (PGY) 1, 2, 3, or 4), gender, and age group (up to and including 29 years and 30 years or older). The age split was chosen based on the published EQ-i 2.0© reference data.^18^ Differences between the four PGYs were analyzed using ANOVA followed by post-hoc testing for linear relationships between PGY and EI score. Gender differences and age group differences between overall EI and between each of the subcategories were analyzed using an independent two-sample t-test with Welch’s correction to account for unequal sample sizes and assuming unequal variances. Differences were considered significant if p<0.05.

## Results

### Demographics

All 35 residents (23 women and 12 men) completed the EQ-i 2.0© (100% response rate). There were 11 participants from PGY-1 (31%), nine from PGY-2 (26%), nine from PGY-3 (26%), and six from PGY-4 (17%). Fourteen participants were over 30 years old (40%) and 21 were under 30 (60%).

### Overall EI scores of general pediatric residents

**Figure 1:**
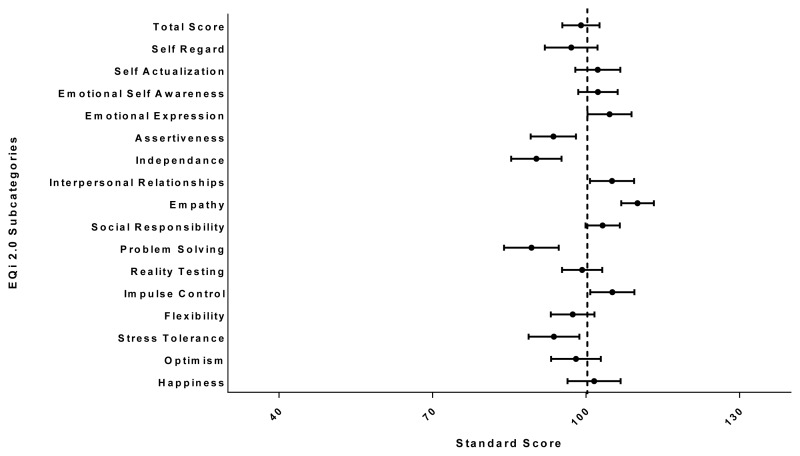
Pediatric resident scores by subcategory. The mean score for each subcategory is represented by a circle. The dashed line represents the professional normative mean. The error bars represent the 95% confidence interval.

No difference was found between the overall EI score of residents (M=99, SD=10.58) and the normative professional sample mean (M=100, SD=15) (p=0.57). When the pediatric residents’ overall EI score was compared to the general population normative sample, their scores were higher (M=107.6, SD=10.58 vs. M=100, SD=15) (p=0.002). However, resident scores were lower than the professional sample means (M=100) in the subcategories of Stress Tolerance (M=93.7, 95% CI 88.8, 98.7), Assertiveness (M=93.6, 95% CI 89.1, 98), Independence (M=90.2, 95% CI 85.3, 95.2), and Problem Solving (M=89.3, 95% CI 84, 94.7) and higher than expected in the areas of Emotional Expression (M=104.3, 95% CI 100.3, 108.9), Interpersonal Relationships (M=105.1, 95% CI 100.7, 109.4), Empathy (M=110.1, 95% CI 106.9, 113.2), and Impulse Control (M=105.1, 95% CI 100.8, 109.4) (Figure 1).

### Differences by postgraduate year of training

There were no statistically significant differences between postgraduate years in overall score (p=0.74) (Table 1). A statistically significant difference was found between postgraduate years in the subcategory Assertiveness (F(3,31)=3.5, p=0.03). Post-hoc testing was statistically significant (p<0.05) for a linear relationship between increasing postgraduate year and increasing Assertiveness score (r^2^=0.24). There were no significant differences in any other composite or subcategory scores between post-graduate years (Table 2).

### Differences by gender

There were no significant differences in overall scores between women (M=100.5, SD=9) and men (M=96.0, SD=13.08). The only significant difference was in the subcategory of Emotional Expression with women (M=108.6, 95% CI 103.7, 113.7) outperforming men (M=96.7, 95% CI 89.8, 103.5).

### Differences by age

There was no difference between the overall EI scores between the two age groups [M=101.6 vs. 97.3, (p=.22)]. However, the older age group achieved higher scores compared to the younger group for the subcategories Independence [(M=98.6, 95% CI 92.1, 105) vs. (M=84.8, 95% CI 78.5, 91)] and Problem Solving [(M=97.9, 95% CI 91.6, 104.2) vs. (M=84.2, 95% CI 77, 91.4)].

## Discussion

This study was able to demonstrate areas of relative strength and weakness in EI amongst general pediatric residents working at a relatively large pediatric hospital in Western Canada. The overall EI of pediatric residents was not significantly different from a normative group of college-educated professionals, but, as anticipated, was higher than the general population norms. Pediatric residents scored the strongest in the subcategories *Emotional Expression, Interpersonal Relationships, Empathy, and Impulse Control* in relation to the professional normative scores. There was no correlation between overall EI and year of training. There was a positive correlation between increasing year of training and increasing *Assertiveness*. Areas of relative weakness were in the subcategories *Stress Tolerance, Assertiveness, Independence*, and *Problem Solving*. There were statistically significantly higher scores in the subcategories *Independence* and *Problem Solving* for residents over 30 years of age as compared to their younger colleagues. Women outperformed men in the subcategory *Emotional Expression.*

It is surprising that more subcategories did not improve as residency training advanced, such as *Independence,* since residents at senior levels are given graduated responsibility in leading teams. However, this is consistent with previous descriptive studies by Chan et al. and Jensen et al. with general surgery residents.^14^,^15^ This potentially suggests that trainees may succeed in a pediatric residency based on their previously learned EI skills rather than acquiring these skills during residency. As Jensen et al. suggest, the EI of individuals within this select group may have developed sooner than normal due to intense life or educational experiences^15^ in first year of residency or medical school or before, then levelled off throughout the rest of residency. It may also be that growth in EI is stunted during an intense residency training program, due to decreased *Optimism, Stress Tolerance,* or an increased dependence on preceptors or other residents.^15^ It does seem likely that some EI-based skills do increase with aspects of training since *Assertiveness,* a skill honed with increasing responsibilities such as supervising junior trainees, does show a positive linear correlation in our study.

The high degree of empathy for others exhibited by pediatric residents may be a constraint when making decisions and in leading. Mnoonkin et al. suggest that empathy and assertiveness function as two independent dimensions of negotiation behavior that interact with each other in predictable patterns.^21^ The interaction between empathy and assertiveness results in descriptions of styles of negotiation such as “the competitor,” who displays substantial assertion but little empathy, and an “accommodator,” who displays substantial empathy but little assertion.^21^ When both empathy and assertiveness are lacking, an “avoidant” style is the result.^21^ Further development of leadership skills by exploring the interactions between empathy and assertiveness in small groups, as suggested by Mnoonkin et al., could allow residents to understand their patterns of interaction. These small-group reflection activities may be a strategy to allow residents to gain competence in areas of the CanMEDS framework published by the Royal College of Physicians and Surgeons of Canada which are more difficult to teach in large group lecture formats. The CanMEDS framework, which includes seven roles (Medical expert, Communicator, Collaborator, Leader, Health advocate, Scholar and Professional), identifies and describes the abilities physicians require to meet the health care needs of the community they serve and provides structure to residency training programs across Canada.^22^ Providing a greater understanding of patterns of interaction to residents, in the context of their own skills, may improve their effectiveness in negotiating and counselling patients, hone skills in the CanMEDS framework, as well as improve their overall EI score.

Postgraduate trainee’s emotional intelligence has been studied in selected groups in the past using various measurement instruments. We were interested if comparisons could be drawn between these groups. When comparing pediatric residents to other residency programs, general surgery residents also scored above the population average on the EQ-i©, however orthopedic residents scored below average on the Mayer-Salovey-Caruso Emotional Intelligence Test (another EI psychometric instrument).^14^,^15^ A study of anesthesia residents demonstrated that many of the subscales of resident performance assessed in the United States by the Accreditation Council for Graduate Medical Education criteria are correlated with EI, however scores were not available for comparison with our study.^19^,^23^ Recently, first year pediatric interns in the United States were assessed by the Emotional and Social Competency Inventory, a multi-source feedback EI measure, which showed scores in all categories above population norms.^17^ These scores were generally high, with little variance amongst subcategories, and are not comparable to the EQ-i 2.0©.

The lack of a difference between men and women in overall EI is consistent with population norms.^15^ However, it is surprising that the only gender difference in the subcategories was *Emotional Expression* with women out-performing men. Subcategories in which women scored higher than men in the population norms include *Emotional Self-awareness, Interpersonal Relationships, Empathy*, and *Social Responsibility* while men scored higher in *Stress Tolerance* and *Problem Solving*.^18^ It is possible that our sample was too small to elicit these differences. Other explanations would be that these gender differences do not apply to those who enter pediatric residency, or perhaps and hopefully, pediatric training minimizes these differences.

Previous research found that EI increases with age although we found no statistically significant differences in our study. This may be due to insufficient sample size and a narrow age range. The subcategories of *Independence* and *Problem Solving* were statistically significantly higher however, indicating some consistency with the general population.

Identifying relative strengths and weakness in EI is important, but not likely to enhance the physician-patient relationship without further intervention training. As discovered by Reed et al. when studying residents in their first year of training high EI scores were not correlated with an increased ability to deliver bad news.^24^ They concluded that increased resident EI does not replace specific training in the delivery of bad news.^24^ In the design of EI-related learning sessions, a systematic review by Cherry et al. provided suggestions for the structure of this training. Their study reviewed the impact of structured education sessions to enhance EI in medical students and found a small positive effect on student attitudes and knowledge towards the impact of EI on their patients; unfortunately, they noted that the EI interventions they were studying did not have much impact on overall behavioural change.^7^ No similar study has been published on post-graduate trainees to date. Cherry et al. suggested that the most impactful interventions were delivered over a short span of time (less than one month), to students later in their educational training, and to women.^7^ While these findings may be useful to design a future intervention in postgraduate pediatric education, helping guide residents in an understanding of EI-related competencies, such as helping them understand patterns of interaction and assisting with self-reflection, may also be meaningful. Further research needs to be done in order to ascertain the best way to support a behavioural change from an EI-related intervention.^7^

Limitations to our study, which may affect its validity, include utilizing a convenient (although complete) sample of pediatric residents from only one center. Future studies would benefit from the inclusion of residents from several institutions to improve generalization of results to all of Canada and an increased sample size to provide adequate power for detecting statistically significant results. The self-reporting property of the EQ-i 2.0© may also affect this study’s validity, for example high *Empathy* scores may reflect resident views and not their true skills. Future work may include multi-source feedback to calculate EI scores using tools such as the EQ 360©. The EQ 360© is directly comparable to the EQ-i 2.0© and has the benefit of assessing the same emotional and social skills as the self-reporting EQ-i 2.0© but from multiple observer’s perspectives.

Future research may also study how resident EI scores correlate with patient satisfaction or other patient outcomes. Systematic reviews have demonstrated that although many people are writing opinion pieces on EI and completing descriptive studies, there are actually very few empirical studies linking a doctor’s skill or competence with EI or studies showing how high EI is correlated with improved patient outcomes.^9^,^10^,^24^–^26^ One of the only studies to do this is by Wagner et al. and included 30 residents and family physicians at an academic family medicine institution and only showed the subscale *Happiness,* as measured by the EQ-i©, to be correlated with improved patient satisfaction.^10^ Our descriptive study may be used as a starting point for further investigation into the impact of EI on patients and families cared for by pediatric residents.

### Conclusions

Pediatric residents, as a whole, score higher than the general population norms, but are consistent with scores of other professionals, including general surgery residents, as measured by self-report using the EQ-i 2.0©. Individual score reports vary and can provide powerful individual feedback. Areas of strength overall in pediatric residents were *Emotional Expression, Empathy, Interpersonal Relationships and Impulse Control,* while areas of weakness were the subcategories *Independence*, *Assertiveness*, *Problem Solving*, and *Stress Tolerance.* It may be possible to develop an EI-related learning enhancement session based on the weaknesses noted while continuing to emphasizing individual medical knowledge and skill development.

## Figures and Tables

**Table 1:** Descriptive statistics for postgraduate year groupings overall EI score

	PGY-1	PGY-2	PGY-3	PGY-4
**Number of Participants**	11	9	9	6
**Mean**	96.7	99.1	99.1	102.8
**Standard Deviation**	11.60	12.53	8.62	9.60

**Table 2:** Descriptive statistics for postgraduate year groupings composite and subcategory scores with analysis for differences between group means by one-way ANOVA (^*^statistical significance)

Composite Categories	PGY-1 (mean, standard deviation)	PGY-2 (mean, standard deviation)	PGY-3 (mean, standard deviation)	PGY-4 (mean, standard deviation)	*p-*value as analyzed by one-way ANOVA
Self-Perception	100.9, 14.5	98.9, 10.0	96.8, 9.0	102.5, 9.2	0.76
Self-Expression	88.7, 12.3	96.7, 13.8	100.4, 8.5	102.8, 12.8	0.08
Interpersonal	103.8, 12.1	110.1, 9.4	107.2, 9.1	109, 9.7	0.56
Decision Making	97.8, 12.7	98.4, 13.5	95.4, 13.1	96.7, 15.8	0.97
Stress Management	93.8, 9.1	91.4, 16.1	96.9, 12.1	101.3, 5.1	0.41
**Subcategories**					
Self-Regard	99.9, 16.4	91.1, 17.6	96.3, 11.6	102.0, 13.2	0.50
Self-Actualization	101.8, 16.2	105.4, 14.2	99.1, 9.6	103.2, 9.2	0.78
Emotional Self-Awareness	104.2, 9.5	104.1, 13.8	97.3, 13.2	103.7, 5.3	0.51
Emotional Expression	98.2, 14.4	108.8, 15.5	109.3, 8.5	104.8, 12.1	0.22
Assertiveness	88.8, 8.8	89.0, 16.5	95.7, 10.1	106.2, 9.4	0.03^*^
Independence	85.4, 11.6	90.4, 16.1	92.9, 15.3	95.2, 15.6	0.53
Interpersonal Relationships	102.2, 17.3	104.8, 6.2	105.9, 12.9	109.5, 10.4	0.73
Empathy	108.2, 9.1	114.3, 10.7	107.6, 9.3	110.8, 6.5	0.39
Social Responsibility	98.9, 9.1	105.8, 11.9	105.9, 6.9	103.3, 10.6	0.34
Problem Solving	89.1, 12.0	85.4, 21.7	92.3, 12.7	91.0, 17.3	0.82
Reality Testing	101.7, 14.5	100.0, 7.5	95.4, 10.4	99.2, 13.2	0.69
Impulse Control	104.8, 13.1	111.2, 9.0	101.3, 13.6	102.2, 14.2	0.36
Flexibility	97.2, 12.2	94.2, 16.2	100.3, 11.4	98.0, 9.2	0.79
Stress Tolerance	88.8, 11.7	92.7, 18.4	94.9, 16.5	102.5, 4.4	0.32
Optimism	99.6, 13.9	93.6, 19.6	97.0, 11.3	103.3, 8.5	0.60
